# *Eidophasia assmanni* sp. nov., the first alpine representative of the genus, detected in the Russian Altai Mountains (Lepidoptera, Plutellidae)

**DOI:** 10.3897/zookeys.959.54259

**Published:** 2020-08-14

**Authors:** Peter Huemer, Jae-Cheon Sohn

**Affiliations:** 1 Tiroler Landesmuseen Betriebsges.m.b.H., Sammlungs- und Forschungszentrum, Naturwissenschaftliche Sammlungen, Krajnc-Straße 1, A-6060 Hall in Tirol, Austria Sammlungs- und Forschungszentrum Innsbruck Austria; 2 Department of Science Education, Gongju National University of Education, Woongjinro 27, Gongju, Chungnam 32553, South Korea Gongju National University of Education Gonju South Korea

**Keywords:** DNA barcoding, endemism, new species, Siberia, Yponomeutoidea

## Abstract

*Eidophasia assmanni***sp. nov.**, a new species of Plutellidae from the alpine zone of Russian Altai Mountains, is described from diagnostic morphology and DNA barcodes. Male adult and genitalia are illustrated, whereas the female sex remains unknown. The species inhabits alpine scree with patchy herbaceous plants and is considered as possible endemic species of the Altai Mountains. An updated checklist of the 13 global *Eidophasia* Stephens, 1842 species is provided. The likely polyphyly of the genus is discussed from molecular data of the barcode region of the mt COI gene.

## Introduction

The Lepidoptera fauna from the Altai region attracted attention from lepidopterists early on, e.g., Lederer (1853, 1855), and numerous expeditions have acquired extensive material from the area. However, much of this material remains unpublished and hidden in several institutional and private collections. Whereas larger moths and butterflies are nowadays relatively well known (i.e., Tshikolovets 2009, Volynkin 2012), biogeography (Huemer et al. 2017) and taxonomy of many so-called Microlepidoptera are still insufficiently documented, resulting in several new descriptions from various families during the last years (i.e., Bidzilya et al. 2019, Buchner and Šumpich 2020, Gaedike and Šumpich 2017, Šumpich et al. 2019, 2020, Ustjuzhanin and Kovtunovich 2007).

In this paper a new species of Plutellidae from the Republic of Altai is described. Plutellidae are a moderately diverse family of Yponomeutoidea with ca. 150 described species from 48 genera listed on a worldwide scale (van Nieukerken et al. 2011), and with few taxa added more recently (i.e., Baraniak et al. 2017, Landry and Hebert 2013). The Russian fauna includes 18 species from six genera, with only three species known from the Republic of Altai, two belonging to the genus *Plutella* Schrank, 1802 and one to *Plutelloptera* Baraniak, 2007 (Sinev 2019). The new species is assigned to *Eidophasia* Stephens, 1842, a genus with four representatives in the Russian Federation, two restricted to the Far East, one to the European part of the country, and only one, *E. messingiella* (Fischer von Röslerstamm, 1839), being more widely distributed in the southern parts of the country (Sinev 2019). The latter species is the only representative with a wide Palaearctic distribution pattern, ranging from Western Europe to East Asia (Sohn 2012), including the Middle East (Alipanah 2019).

## Materials and methods

Material of voucher specimens was either pinned and spread or traditionally set. The labels of the holotypes are quoted in their original spelling. Genitalia preparations followed standard techniques (Robinson 1976). DNA barcode sequences are based on a 658 base-pair long segment of the mitochondrial COI gene (cytochrome c oxidase 1). DNA samples (dried legs) were prepared according to the prescribed standards in the Barcode of Life Data Systems (BOLD v. 4.0. http://www.boldsystems.org; Ratnasingham and Hebert 2007). Legs from 52 specimens Plutellidae were successfully processed at the Canadian Centre for DNA Barcoding (CCDB, Biodiversity Institute of Ontario, University of Guelph) to obtain DNA barcodes using the standard high-throughput protocol described in deWaard et al. (2008), supplemented by a further 48 public sequences from BOLD. Obtained sequences range between 522 and 658 bp, with 88 specimens represented by a full DNA barcode. The sequenced material includes 41 specimens of *Eidophasia*, whereas the remaining 59 sequences depict the type species of the outgroup genera *Plutella*, *Plutelloptera*, *Pseudoplutella* Baraniak, 2007, and *Rhigognostis* Staudinger, 1857. Furthermore, an unpublished specimen of *E. albidorsella* (Walsingham, 1881) (specimen ID USNMENT00657823) was considered for analysis. Sequences were submitted to GenBank, and further details including complete voucher data and images can be accessed in the public dataset “DS-EIDOASMA *Eidophasia assmanni* sp.n.” https://dx.doi.org/10.5883/DS-EIDOASMA in BOLD. Degrees of intra- and interspecific variation of DNA barcode fragments were calculated under Kimura 2 parameter model of nucleotide substitution using analytical tools of BOLD systems v. 4.0. A Neighbour-joining tree of DNA barcode data of central and south-eastern European taxa was constructed using MEGA 6 (Tamura et al. 2013) under the Kimura 2 parameter model for nucleotide substitutions.

Identification success was furthermore assessed by the Barcode Index Number (BIN) system as implemented on BOLD (Ratnasingham and Hebert 2013). This system employs a two-stage algorithm that groups all sequences > 500 bp that meet defined quality criteria into Operational Taxonomic Units (OTUs) and automatically assigns new sequences, irrespective of their previous taxonomy and origin. Similarities or differences between BINs and morphological species identification were assessed.

Photographs of the adults were taken with an Olympus SZX 10 binocular microscope and an Olympus E 3 digital camera and developed using the software Helicon Focus 4.3 and Adobe Photoshop CS4 and Lightroom 2.3. Genitalia photographs were taken with an Olympus E1 Digital Camera through an Olympus BH2 microscope.

## Results

### Taxonomic part

#### *Eidophasia* Stephens, 1842

The genus *Eidophasia* Stephens, 1842 was established as an objective replacement name for *Parasemia* Stephens, 1841, a junior homonym of *Parasemia* Hübner, [1820] 1816 (Erebidae) (Nye and Fletcher 1991). Its type species *Parasemia transversella* Stephens, 1841, by monotypy, is currently considered as a junior subjective synonym of *Plutella messingiella* Fischer von Röslerstamm, 1839. For the correct date of description of *P. messingiella* see Rodeland (2018).

The generic definition followed in this paper is largely based on Zagulyaev (1990), with characters of wing venation, particularly the almost parallel and straight veins M1 and M2 in the hindwing, and the bases of R2 and Cu2 at same level or R2 much closer to base in forewing, the second segment of labial palps characterized by a tuft of long scales, the 3^rd^ segment equal or slightly longer than the 2^nd^ segment, and the antennae partially knobby, due to specialised scales. However, as discussed earlier by Baraniak and Sohn (2015), members of the genus are heterogeneous in morphology and no convincing synapomorphies have been proposed for *Eidophasia* to date.

The genus is mainly Holarctic with currently 13 known species. Three species are restricted to North America (*E. dammersi* (Busck, 1934), *E. albidorsella* (Walsingham, 1881), *E. vanella* (Walsingham, 1881)), and one to New Guinea (*E. peristigma* Diakonoff, 1955). Robinson and Sattler (2001) listed 11 species, but meanwhile *E. zukowskyi* Amsel, 1939 and *E. infuscata* Staudinger, 1870 have been upgraded to species level (Baraniak and Sohn 2016, Sohn and Baraniak 2016), whereas the earlier synonymisation of *E. aereolella* Lhomme, 1949 with *E. messingiella* is followed (Gibeaux 1978). *Eidophasia lvovskyi*, a species already mentioned in the Catalogue of Russian Lepidoptera (Sinev 2019), is still unpublished (Sinev in litt.). We divided the species of *Eidophasia* into two informal species groups, based on the differences in the sacculus of male genitalia and the ductus bursae of female genitalia (see Discussion).

### Checklist of *Eidophasia*

***Eidophasia* Stephens, 1842**

*Parasemia* Stephens, 1841 *nec* Hübner, [1820] 1816 (homonym)

*Spania* Guenée, 1845

*Hufnagelia* Reutti, 1853

*Eudophasia* Herrich-Schäffer, 1853 (misspelling)

The *messingiella* species group

***Eidophasia messingiella* (Fischer von Röslerstamm, 1839) (*Plutella*)**

= *transversella* (Stephens, 1841)

= *muellerella* Rougemont, 1903

= *aereolella* Lhomme, 1949

***Eidophasia infuscata* Staudinger, 1870**

***Eidophasia tauricella* Staudinger, 1880**

***Eidophasia albifasciata* Issiki, 1931**

***Eidophasia dammersi* (Busck, 1934) (*Plutella*)**

***Eidophasia albidorsella* (Walsingham, 1881) (*Plutella*)**

***Eidophasia vanella* (Walsingham, 1881) (*Plutella*)**

***Eidophasa assmanni* sp. nov.**

The *syenitella* species group

***Eidophasia syenitella* Herrich-Schäffer, 1854 (*Eudophasia* [sic])**

= *concinnella* Christoph, 1888

***Eidophasia zukowskyi* Amsel, 1939**

***Eidophasia hufnagelii* (Zeller, 1839) (*Plutella*)**

***Eidophasia insulella* (Walsingham, 1900) (*Caunaca*)**

***Eidophasia peristigma* Diakonoff, 1955**

#### Eidophasia assmanni sp. nov.

Taxon classificationAnimaliaLepidopteraPlutellidae

2B328278-3597-5B17-9B16-F542E129FEA3

http://zoobank.org/A204788A-24C2-41A3-8F06-6DABDD5DDF72

**Figure 1.**
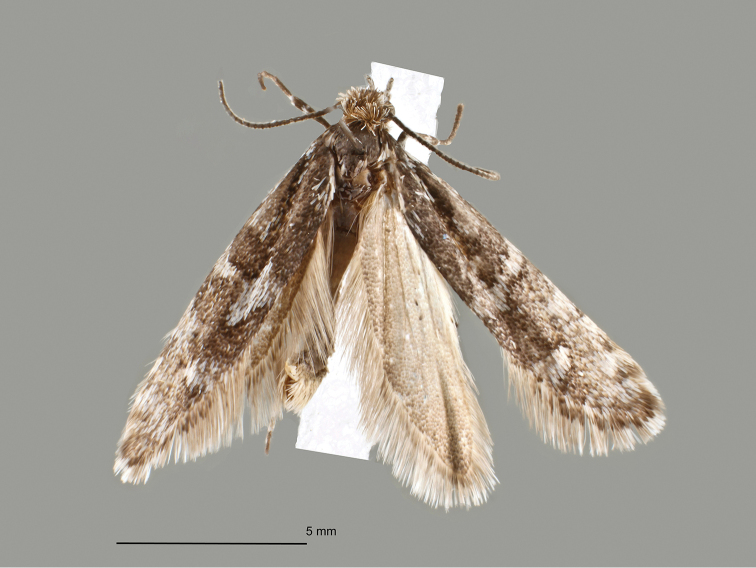
*Eidophasia assmanni* sp. nov., holotype, scale bar: 5 mm.

**Figure 2.**
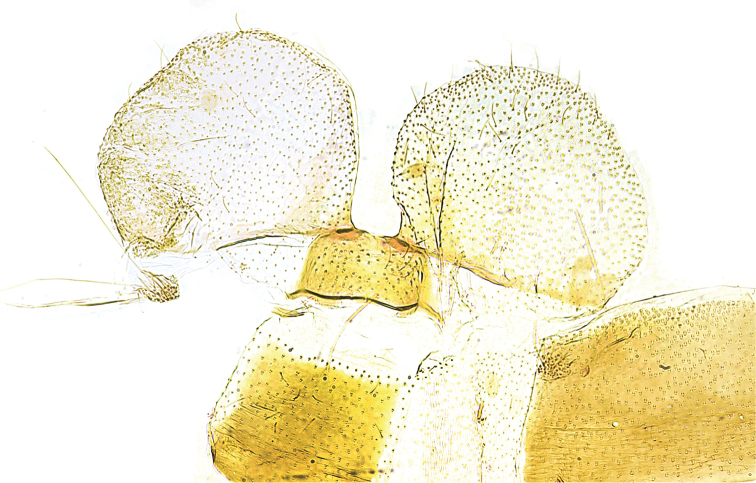
*Eidophasia assmanni* sp. nov., holotype, male pre-genitalia segments.

**Figure 3.**
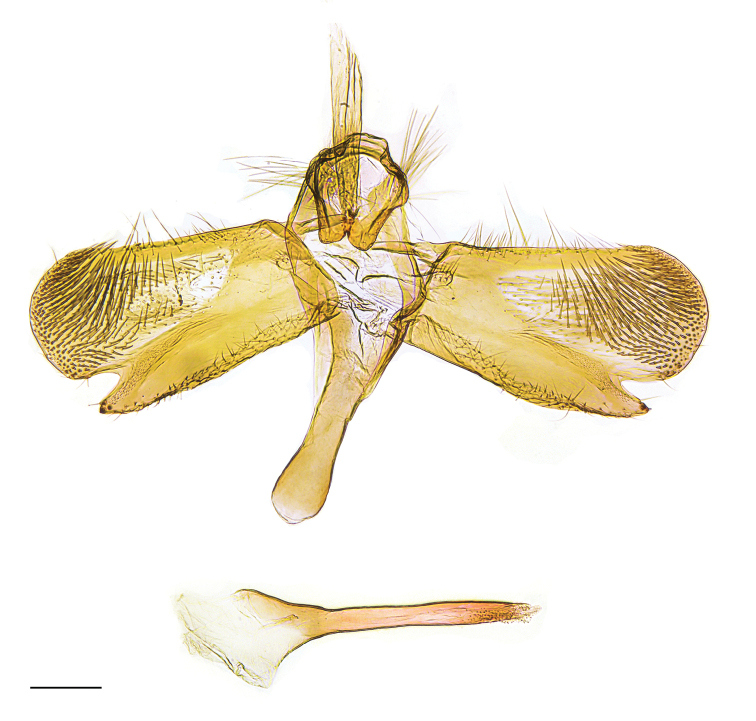
*Eidophasia assmanni* sp. nov., holotype, male genitalia. Scale bar: 0.2 mm.

**Figure 4.**
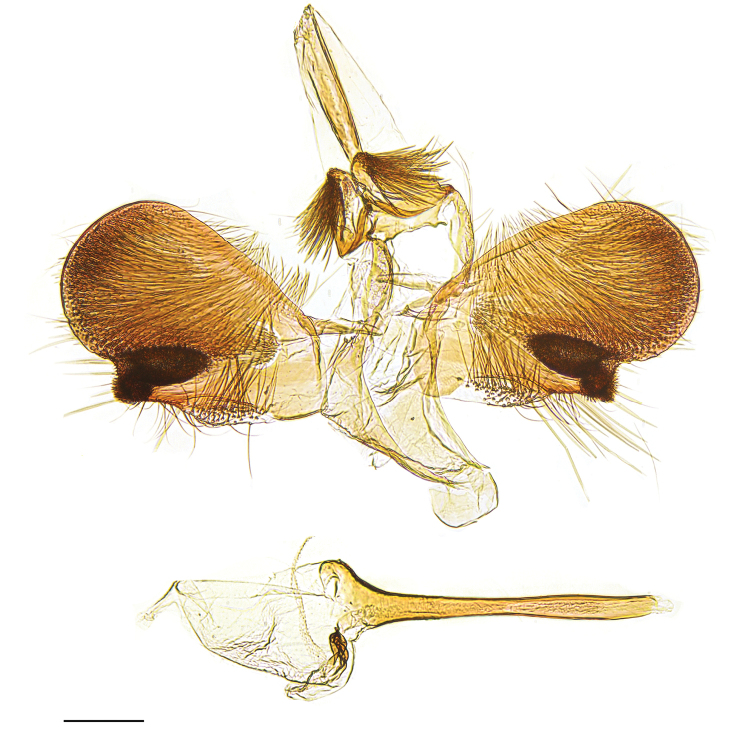
*Eidophasia vanella* (Walsingham, 1881), male genitalia, Canada, gen. slide 20/1586 P. Huemer. Scale bar: 0.2 mm.

**Figure 5.**
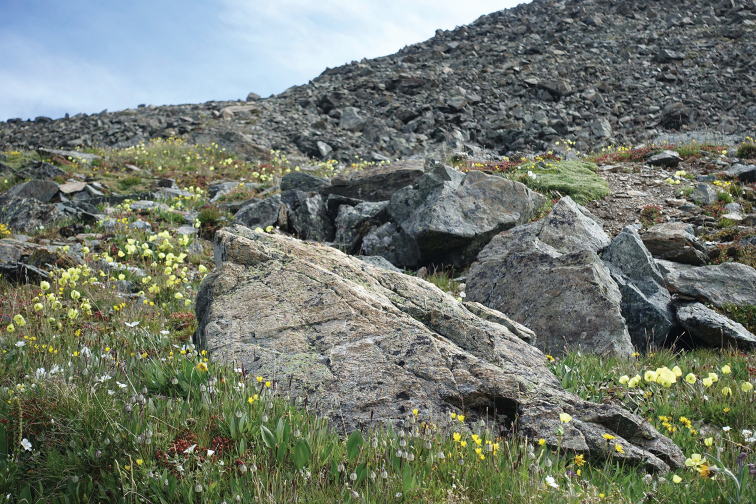
*Eidophasia assmanni* sp. nov., type locality (Russia, Altai Mountains).

**Figure 6.**
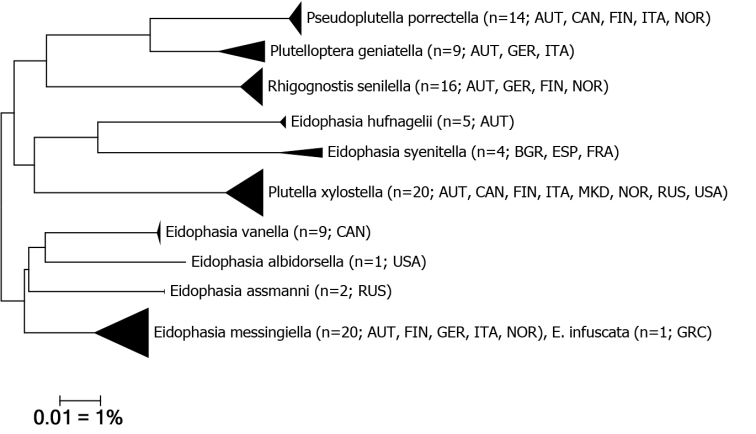
Neighbor-Joining tree (Kimura 2 parameter, built with MEGA 6; cf. Tamura et al. 2013) of *Eidophasia* spp. and type species of selected Plutellidae genera. The width of the triangles represents the sample size, and the depth the genetic variation within the cluster. Source: DNA Barcode data from BOLD (Barcode of Life Database, cf. Ratnasingham and Hebert 2007).

Figures 1 , 2 , 3 , 4 , 5 , 6

##### Material.

***Holotype*** ♂: “Russia, Altai Republic, / Ulagan distr., 10 km NE / Aktash vill., Kuraj Mts. / Range, between rivers / Korumdyajry and Yarlyamry, / 50°20'N, 87°45'E, stone / tundra, 2750–2800 m, / 07.08.2016, leg. Huemer & / Wiesmair, TLMF 2016–020” “DNA Barcode / TLMF Lep 21215” “YPO 162 ♂ P. Huemer” (Tiroler Landesmuseum Ferdinandeum, Innsbruck, Austria). ***Paratype***: 1 ♂, same data as holotype, but 2900–3000 m, 30.vii.2016, DNA Barcode TLMF Lep 20484 (Tiroler Landesmuseum Ferdinandeum, Innsbruck, Austria).

##### Diagnosis.

*Eidophasia assmanni* is unmistakable in habitus due to the inconspicuous wing markings, which are clear and prominent in *E. assmanni*, and white or yellow in all other known species of the genus. The male genitalia are unique in *Eidophasia* by the oblong shape of the valva with straight dorsal and ventral edges and particularly the very long and apically pointed sacculus with largely reduced spiniform setae on distal end. In *E. messingiella* and related taxa the valva is obovate with a curved sacculus and sets of spiniform setae at apex (see Sohn and Baraniak 2016), whereas in *E. vanella* the sacculus is very short with large sets of spiniform setae and with a pair of brush-like coremata on the outer side of the valva (Figs 3, 4). DNA barcodes show a minimum distance > 5% to the nearest species *E. vanella* and *E. messingiella*. *Eidophasia assmanni* so far is the only known species of the genus restricted to an alpine habitat.

##### Description

(Fig. 1). Forewing length (from base to apex): 6.0–6.4 mm. Head dark grey-brown, sparsely intermixed with white scales, particularly at vertex and lateral part of frons; labial palpus mixed grey-brown and white, particularly outer surface of 1^st^ and second segment predominantly white, second segment with short ventral tuft of scales; first and second segment about the same length, third segment much longer, upcurved; antenna dark grey-brown, with weak white-grey annulation. Thorax dark grey-brown, patagia with few white-grey scales, tegula with some white scales; fore and mid-legs dark grey-brown on upper surface, distal end of tarsal segments weakly ringed white-grey, ventral surface predominantly white-grey, hindlegs predominantly white-grey; forewing dark grey-brown with weakly delineated white-grey markings: few scales at base, indistinct narrow transverse antemedian line, costal spot at two-thirds, irregularly delineated costal and tornal spots at about four-fifths, and extended white-grey mottling in distal third; fringe white-grey, with dark grey-brown basal line; hindwing light grey-brown, fringe white-grey with grey-brown basal line; underside of fore and hindwing, white-grey, without pattern. Abdomen grey-white, lighter at ventral surface.

Pre-genital segments (Fig. 2). Tergite VIII small, sub-rectangular, posterior-laterally with large semi-oval appendage, covering parts of genitalia capsule laterally; brush of long coremata in intersegmental membrane of segment VII and VIII, extending to about posterior margin of appendages.

Male genitalia (Fig. 3). Tegumen semi-elliptical; tuba analis slightly longer than tegumen, slender, weakly sclerotized; teguminal process prominent, with straight outer edge and broadly rounded apex, basal part setose; valva oblong, nearly three times length of basal width, dorsal and ventral edges straight, gradually widened towards semi-oval apex, membranous distomedial part from about middle of valva expanded to apex and densely covered with setae; sacculus nearly extended to apex of valva, sclerotized ventral margin straight, inner side with short setae, apically pointed with two to three minute spiniform setae; vinculum sub-triangular; saccus about three-quarters length of valva, massive, distal third weakly dilated, apex rounded; phallus about length of valva, straight, basally bulbous, vesica with small group of microtrichia apically (uneverted vesica).

##### Molecular data.

BIN: BOLD:ADE0025. The intraspecific average distance of the barcode region is 0% (n = 2), the minimum distance to the Nearest BIN in BOLD, *E. vanella*, is 5.25% (p-distance), whereas the Nearest Neighbour in our dataset is a specimen of *E. messingiella* with 5.88% divergence (Table 1).

**Table 1.** Intraspecific mean K2P (Kimura 2 Parameter) divergences, maximum pairwise distances, and distance to Nearest Neighbour in *Eidophasia* and generic type species of related genera.

Species	Mean Div.	Max Div.	Nearest Species	Nearest Neighbour	Distance to NN
*Eidophasia assmanni*	0	0	*Eidophasia messingiella*	CGUKD020-09	5.88
*Eidophasia albidorsella*	N/A	0	*Eidophasia vanella*	LPAB805-08	6.24
*Eidophasia hufnagelii*	0.22	0.32	*Eidophasia syenitella*	LEFIJ2890-15	9.59
*Eidophasia infuscata*	N/A	0	*Eidophasia messingiella*	LEFIE702-10	0.77
*Eidophasia messingiella*	0.48	2.99	*Eidophasia infuscata*	LEFIJ2893-15	0.77
*Eidophasia syenitella*	1.76	2.18	*Eidophasia hufnagelii*	LEATJ1474-16	9.59
*Eidophasia vanella*	0.03	0.15	*Eidophasia messingiella*	CGUKD020-09	5.44
*Plutelloptera geniatella*	0.67	2.5	*Pseudoplutella porrectella*	LEATJ1471-16	5.83
*Pseudoplutella porrectella*	0.18	0.77	*Plutelloptera geniatella*	PHLAA575-09	5.83
*Plutella xylostella*	0.68	2.18	*Eidophasia messingiella*	CGUKD020-09	9.07
*Rhigognostis senilella*	0.52	1.24	*Eidophasia messingiella*	CGUKD020-09	8.62

##### Bionomics.

The host plant and early stages are unknown. Though it seems possible that the species shows similar behaviour to other *Eidophasia* spp., with a host plant restriction to Brassicaceae; it may also be polyphagous such as the related *E. vanella*. The two adults were found between late July and early August when they were netted during daytime in strong wind conditions. The type-locality is an alpine tundra dominated by rock and scree with patchy herbaceous vegetation (Fig. 5).

##### Distribution.

The species is currently only known from the type locality in the Altai Mountains (Altai Republic, Russian Federation).

##### Etymology.

The species is dedicated to the Director of Tyrolean Federal State Museums Mag. Dr. Peter Assmann to his 55^th^ birthday and in recognition of his particular support of Natural History Collections already in his former and present career.

### Molecular analysis

A DNA barcode gap analysis of seven analysed species of *Eidophasia* (Table 1, Fig. 6) proves a high barcode divergence, ranging from a minimum of 5.44% to 9.59%, with the exception of *E. messingiella* and the recently separated *E. infuscata* (Sohn and Baraniak 2016) which only show a 0.77 % divergence. However, these two allopatric species show only weak diagnostic characters in morphology and may be considered as subspecies. The divergence of *E. assmanni* to the Nearest Neighbour *E. messingiella* is 5.88%. In comparison, the intraspecific divergence in *Eidophasia* species is low, and > 2% only in *E. messingiella* (based on a deviating specimen from northern Italy) and *E. syenitella* (Table 1).

The interspecific divergence of *E. messingiella* to the type species of *Plutella* and *Rhigognostis* is ca. 9% (Fig. 6). This extent is similar to the interspecific divergences within *Eidophasia*, hinting the non-monophyly of the genus (see Discussion for further evidence).

## Discussion

As already discussed by Landry and Hebert (2013), the generic limits in *Plutella* and its allied genera need further assessment. This is particularly relevant for the genus *Eidophasia* which in its current scope is most likely polyphyletic as indicated by its heterogeneous morphology (Baraniak and Sohn 2015) and the molecular data of the DNA barcode region (Landry and Hebert 2013, this study). The barcoded species of the genus *Eidophasia* were divided into two clusters. One cluster (the *messingiella* species group) included the type species of the genus, *E. messingiella*, and three congeners: *E. vanella*, *E. albidorsella*, and *E. assmanni*. The other cluster (the *syenitella* species group) included two species of *Eidophasia*, *E. syenitella* and *E. hufnagelii*. The former cluster shows two major differences in genital features from the latter: the distal part of sacculus of the valva flapped in various degrees, and the ductus bursae entirely membranous except the antrum. The wing patterns and the genital features of *E. insulella* and *E. peristigma* suggest that they also belong to the *syenitella* species group. The *syenitella* species group may represent a separate genus from *Eidophasia* but their generic assignment is pending in this study.

The DNA barcode sequences of *E. assmanni* clearly show that it belongs to the *messingiella* species group. The *messingiella* species group exhibits considerable interspecific barcode divergence which should be further assessed in the future in an integrative taxonomic study on a worldwide scale.

## Supplementary Material

XML Treatment for Eidophasia assmanni
